# Neuroendocrine Carcinoma of the Urinary Bladder: CT Findings and Radiomics Signature

**DOI:** 10.3390/jcm12206510

**Published:** 2023-10-13

**Authors:** Andrea Coppola, Tonia Gatta, Giacomo Maria Pini, Giorgia Scordi, Federico Fontana, Filippo Piacentino, Roberto Minici, Domenico Laganà, Antonio Basile, Federico Dehò, Giulio Carcano, Francesca Franzi, Silvia Uccella, Fausto Sessa, Massimo Venturini

**Affiliations:** 1 Diagnostic and Interventional Radiology Unit, Circolo Hospital, ASST Sette Laghi, 21100 Varese, Italy; tgatta@studenti.uninsubria.it (T.G.); federico.fontana@uninsubria.it (F.F.); filippo.piacentino@asst-settelaghi.it (F.P.); massimo.venturini@uninsubria.it (M.V.); 2 Department of Medicine and Technological Innovation, Insubria University, 21100 Varese, Italy; federico.deho@asst-settelaghi.it (F.D.); giulio.carcano@uninsubria.it (G.C.); francesca.franzi@asst-settelaghi.it (F.F.); fausto.sessa@uninsubria.it (F.S.); 3 Department of Pathology, Azienda Ospedaliera SS Antonio e Biagio e Cesare Arrigo, 15121 Alessandria, Italy; giacomo.pini@ospedale.al.it; 4 Postgraduate School of Radiology Technician, Insubria University, 21100 Varese, Italy; gscordi@studenti.uninsubria.it; 5 Radiology Unit, Department of Experimental and Clinical Medicine, University Hospital Mater Domini, Magna Graecia University of Catanzaro, 88100 Catanzaro, Italy; robertominici@tiscali.it (R.M.); domenico.lagana@unicz.it (D.L.); 6 Radiodiagnostic and Radiotherapy Unit, Department of Medical and Surgical Sciences and Advanced Technologies, University Hospital “Policlinico-Vittorio Emanuele”, 95123 Catania, Italy; basile.antonello73@gmail.com; 7 Urology Unit, CircoloHospital, ASST Sette Laghi, 21100 Varese, Italy; 8 General, Emergency and Transplant Surgery Unit, Circolo Hospital, ASST Sette Laghi, 21100 Varese, Italy; 9 Patology Unit, Circolo Hospital, ASST Sette Laghi, 21100 Varese, Italy; 10 Pathology Unit, Department of Biomedical Sciences, Humanitas University, Via Rita Levi Montalcini 4, 20090 Pieve Emanuele, Italy; silvia.uccella@hunimed.eu

**Keywords:** neuroendocrine tumours, urinary bladder neoplasms, radiomics, multidetector computed tomography, literature review

## Abstract

**Background**: We present a case series of Neuroendocrine Carcinoma of the Urinary Bladder (NECB) to analyse their radiologic appearance on CT, find a “Radiomic signature”, and review the current literature. **Methods**: 14 CT cases of NECB were reviewed and compared with a control group of 42 patients with high-grade non-neuroendocrine bladder neoplasm for the following parameters: ring enhancement; implantation site; dimensions; density; margins; central necrosis; calcifications; number of lesions; wall thickness; depth of invasion in the soft tissue; invasion of fat tissue; invasion of adjacent organs; lymph-node involvement; abdominal organ metastasis. To extract radiomic features, volumes of interest of bladder lesions were manually delineated on the portal-venous phase. The radiomic features of the two groups were identified and compared. **Results**: Statistical differences among NECB and control group were found in the prevalence of male sex (100% vs. 69.0%), hydronephrosis (71.4% vs. 33.3%), mean density of the mass (51.01 ± 15.48 vs. 76.27 ± 22.26 HU); product of the maximum diameters on the axial plane (38.1 ± 59.3 vs. 14.44 ± 12.98 cm2) in the control group, trigonal region involvement (78.57% vs. 19.05%). About the radiomic features, Student’s *t*-test showed significant correlation for the variables: “DependenceNonUniformity” (*p*: 0.048), “JointAverage” (*p*: 0.013), “LargeAreaLowGrayLevelEmphasis” (*p*: 0.014), “Maximum2DDiameterColumn” (*p*: 0.04), “Maximum 2DDiameterSlice” (*p*: 0.007), “MeanAbsoluteDeviation” (*p*: 0.021), “BoundingBoxA” (*p*: 0.022) and “CenterOfMassB” (*p*: 0.007). **Conclusions**: There is a typical pattern (male patient, large mass, trigonal area involvement) of NECB presentation on contrast-enhanced CT. Certain morphological characteristics and encouraging results about Radiomic features can help define the diagnosis.

## 1. Introduction

Neuroendocrine carcinoma of the urinary bladder (NECB) is a very aggressive and extremely rare cancer, with an annual incidence lower than 1–9/1,000,000, and often diagnosed at an advanced stage. Most patients are Caucasian males in the 6–7th decade of age. The main risk factors include smoking habits and exposure to carcinogenic materials such as industrial dyes [1].

Two typologies are currently recognized: small cell (SCC) and large cell carcinoma (LCC). NECB is often associated with other histological forms of bladder cancer: transitional cell carcinoma, adenocarcinoma, and squamous cell carcinoma [2].

Clinical features of NECB are similar to those of transitional carcinoma of the urinary system and reflect the location and characteristics of the tumour mass. The main symptom is haematuria, reported in 63–88% of cases, which can commonly be associated with dysuria. Urinary obstruction, hydronephrosis, pelvic pain, and urinary tract infection are less frequently present [3].

Diagnosis and staging are classically based on CT study of the mass, pelvic nodes, systemic involvement, and pathological data collected from cystoscopy and transurethral resection of the bladder (TUR-B). Immunochemical staining is useful for establishing the diagnosis [4,5].

Differential diagnosis is with bladder invasion of prostatic SCC, metastatic SCC of another origin, usually from the lung, and primary bladder lymphoma [6]. The staging system used is TNM-staging of transitional cell carcinoma of the bladder [7].

Most cases are diagnosed at an advanced stage with an invasion of perivisceral fat or with lymph node involvement and more advanced metastatic localizations [1].

In those cases where a curative approach is possible, treatment is usually a combination of radical resection, chemotherapy, and radiotherapy [3]. The prognosis of the disease is poor, especially in cases of pure SCC; most of the patients die after a few months from the diagnosis, mainly due to the metastatic progression [8].

Furthermore, the concept of Radiomics has recently been proposed as a non-invasive technique capable of extracting important quantitative features from medical images that are not directly perceptible to the human eye. The extraction of radiomic features from given volumes of interest (VOI) allows the construction of “radiomic signatures”, useful for the prediction of important clinical endpoints such as response to therapy, tumour histology, and overall survival. The application of powerful mathematical algorithms (Machine Learning and Deep Learning) also allows the construction of models with diagnostic, prognostic and predictive potential with respect to different diseases [9].

Scientific studies have shown that radiomics, combined with machine learning techniques, has great potential for managing and following up bladder cancer, with many successful cases [10].

To our knowledge, a small number of NECB cases are reported in medical literature and imaging of this rare disease is poorly described with non-univocal data. The objectives of this study were to report 14 cases of NECB, analysing their radiologic appearance on CT in order to identify a “radiomic signature” of a bladder neuroendocrine tumour (NECB) in contrast-enhanced CT for the purpose of early typing of such lesions and to review the current literature.

## 2. Materials and Methods

### 2.1. Case Series

A query from our Institutional pathology archive was performed retrospectively from September 2021 to January 2010. All pathology slides collected from cystoscopy and TUR-B were retrospectively reviewed by a genitourinary pathologist (S.U.) to confirm the diagnosis. The diagnosis of NECB was made by the WHO classification system [11]. As a control group from the same dataset, a group who had aggressive transitional bladder cancer was selected (inclusion criteria for the control group: Grade 2 or 3; pT 3 or 4).

Clinical histories of these patients were reviewed and pre-therapy contrast-enhanced computed tomography (CECT) scans were sought on our picture archiving and communication system. Data of those patients were retrieved, and sex, age, and presenting symptoms were registered for the present study.

### 2.2. Image Data Acquisition and Interpretation

All CECT were acquired in—at least—basal, portal-venous (fixed 90-s delay), and pyelographic phases after the administration of 1.4 mL/kg of a 350–370 mgI/mL contrast medium (Ioexol, Omnipaque, GE Healthcare, Chicago, IL, USA; Iobitridol, Xenetix, Guerbet; Iopamidol, Iopamiro, Bracco, Milano, Italy). Many different CT scanners were used (Somatom Definition 40, Siemens Healthineers, Erlangen, Germany; Aquilion 64, Toshiba, Tokyo, Japan; Revolution Evo 64, GE; IQon Spectral CT, Philips, Amsterdam, The Netherlands). In all cases, the CECT protocol was 120 KVp, automatic tube current modulation, and slice thickness 0.625–1.5 mm.

A radiologist (A.C.) and a radiology resident (T.G.), with 9 and 3 years of experience in urogenital radiology, reviewed all scans for the following parameters: ring enhancement; implantation site on the bladder wall; dimensions; density; margins; central necrosis; calcifications; the number of wall bladder lesions; wall thickness; depth of invasion in the soft tissue; invasion of the surrounding fat tissue; invasion of adjacent organs; lymph-node involvement; abdominal organ metastasis.

### 2.3. Volume of Interest (VOI) Segmentation

**Figure 1:**
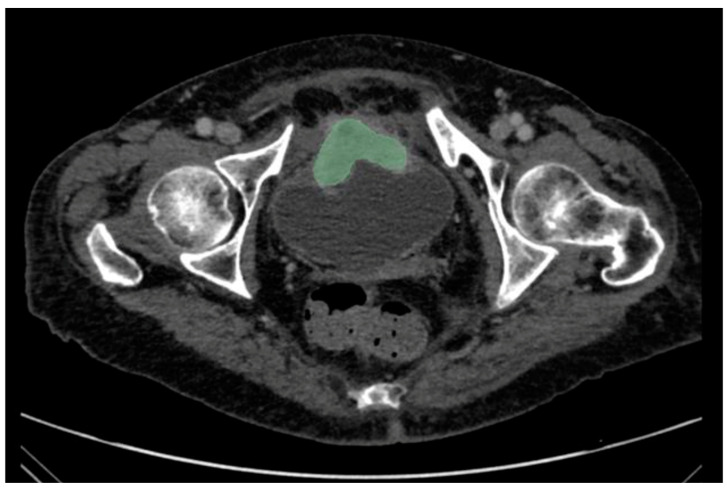
Manual segmentation was performed using 3D Slicer v. 5.0.3 software of a neuroendocrine bladder lesion in an 81-year-old subject on a contrast-enhanced CT scan.

Only the index bladder tumour was considered for evaluation. For the patients with multiple lesions, we selected the largest one on the axial plane. For tumour segmentation, the ROIs of index bladder cancer were manually delineated along the edges of the lesion on each slice for the whole tumour by the same two readers and a trained technician (G.S.) with a free open-source software package (3D Slicer v. 5.0.3; www.slicer.org, accessed on 29 September 2023) on the portal-venous phase (Figure 1). All tumour VOIs were first outlined by two readers independently. Then, they checked together on the outline of the contours for each patient to make a consensus.

### 2.4. Radiomics Feature Extraction

A total of 130 radiomics features were extracted utilizing the SlicerRadiomics extension package (PyRadiomics v. 3.1.0; https://github.com/radiomics/pyradiomics, accessed on 29 September 2023). The radiomics features included 14 shape-based features, 18 first-order statistical features, and 75 texture features (Supplementary Material Files S1 and S2).

### 2.5. Literature Review

PubMed and Web of Science databases were used to search for articles in English published until April 2023. Search terms used were: (“Bladder Neuroendocrine carcinoma” OR “Bladder Small cell carcinoma”) AND (“CT” OR “imaging”). The same data reported for the presented case series were searched and presented.

### 2.6. Statistical Analysis

Descriptive statistics were obtained for all variables assessed in the study population. Mean and standard deviation (SD) are provided for normally distributed variables, median and interquartile range (IQR) are provided for non-normally distributed variables, and number and percentage are provided for categorical variables. Normality was assessed by the Shapiro–Wilk test.

To ensure data consistency, the enrolment ratio for the control group was fixed at 1:3. Groups were compared with parametric or nonparametric tests, according to data distribution, for continuous variables, and with Pearson’s χ^2^ test (Fisher exact test where appropriate) for categorical variables. In all cases, two-tailed tests were used. SPSS v25.0 (IBM, Armonk, NY, USA) was used for all statistical analyses. *p*-values were considered significant when <0.05.

## 3. Results

From September 2021 to January 2010, twenty-one patients with pathological diagnoses of NECB were found. Seven cases were excluded due to a lack of appropriate pre-treatment imaging (non-available in four cases, only basal scan in one case, and artefacts from hip prosthesis in two cases). In the end, 14 pre-treatment contrast-enhanced CT (CECT) suitable for the study were identified. In the control group, 42 cases were enrolled.

In the NECB group, there were 14 men and no women, with a mean age of 76.5 ± 8.7 years (mean ± SD; range: 56–85). Otherwise, in the control group, there were 13/42 women; the mean age was 78.66 ± 6.60 years (mean ± SD; range: 67–90). Student’s *t*-test showed no significant difference (Table 1).

About presentation, there was a statistically significant difference in the rate of hydronephrosis, which was present in 10/14 (71.43%) cases in the NECB group vs. 14/42 (33.33%) in the control group (*p*-value: 0.027). No statistically significant difference was found for the occurrence of haematuria, which was present in 13/14 cases (92.86%) in the NECB group vs. 7/42 (16.67%) in the control group and LUTS, which were present in 5/14 cases (35.71%) in the NECB group vs. 19/42 (45.24%) in the control group.

At image analysis, there was a statistically significant difference in the mean density of the mass (measured by a circular ROI traced on the maximum solid area of the tumour), which was 51.01 ± 15.48 HU in the NECB group vs. 76.27 ± 22.26 HU in the control group (*p* < 0.001). Also, the product of the maximum diameters on the axial plane was 38.1 ± 59.3 cm^2^ (mean ± SD; range: 3.9–190.3) in the NECB group vs. 14.44 ± 12.98 cm^2^ (range: 1.76–72.25) in the control group (*p*: 0.033). The trigonal region was significantly more interested in the NECB group (11/14—78.57% of cases) than in the control group (8/42—19.05%), with a *p*-value of 0.0001 at the Fisher exact test.

No statistically significant difference was found in the finding of ring enhancement, which was present in 9/14 cases (64.29%) in the NECB group vs. 28/42 (66.67%) in the control group, of ill-defined margins (10/14—71.43% vs. 29/42—69.05%), intra-mass necrosis (2/14—14.29% vs. 11/42—26.19%); calcifications (1/14—7.14% vs. 2/42—4.76%); cT3 (3/14—21.43% vs. 33/42—78.57%); cT4 (5/14—35.71% vs. 22/42—52.38%); lymph node involvement (5/14—35.71% vs. 23/42—54.76%); distant metastasis (4/14—28.57% vs. 10/42—23.81%). A comparison between descriptive statistics of the case series and of the control group can be found in Table 2.

Regarding radiomics features, Student’s *t*-test demonstrated a statistically significant difference for the variables “DependenceNonUniformity” (*p*: 0.048), “JointAverage” (*p*: 0.013), “LargeAreaLowGrayLevelEmphasis” (*p*: 0.014), “Maximum2DDiameterColumn” (*p*: 0.04), “Maximum2DDiameterSlice” (*p*: 0.007), “MeanAbsoluteDeviation” (*p*: 0.021), “BoundingBoxA” (*p*: 0.022), and “CenterOfMassB” (*p*: 0.007).

From the literature review, after removing duplicates, a total of 14 articles were included (5 case series and 9 case reports), thus obtaining a total number of 85 patients [12,13,14,15,16,17,18,19,20,21,22,23,24,25]. The demographic, imaging and clinical findings of the 99 patients (14 from the presented case series and 85 from the literature) are shown in Table 1. 

**Figure 2:**
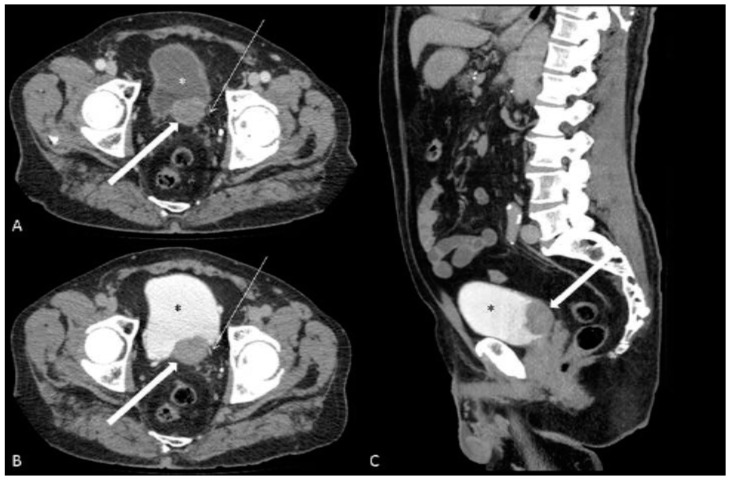
(**A**–**C**) CT urography of neuroendocrine carcinoma of the urinary bladder in an 83-year-old man presenting for haematuria. Axial portal venous phase (**A**), axial (**B**) and sagittal (**C**) excretory phase, demonstrate the presence of a lesion (**thick arrow**) of the posterior wall and trigonal region of the bladder (*). Please note the ring enhancement and the absence of necrosis and calcification. The left ureter (**thin arrow**) runs immediately lateral to the lesion; its appearance is unremarkable.

The patients were more frequently male (100.0%vs. 83.5%) with a mean age of 76.5 ± 8.7 vs. 64.4 ± 6.8 years (mean ± SD). Gross haematuria was the most common symptom in both the case series and the pooled data (100% vs. 70.4%, respectively). Hydronephrosis (71.4% vs. 57.5%), ring enhancement (64.3% vs. 64.7%), ill-defined margins (71.4% vs. 68.4%) and trigonal region involvement (78.5% vs. 56.7%) were the most common findings. Necrosis (14.3% vs. 24.3%) and calcification (7.1% vs. 5.4%) were rarer (Figure 2). 

The density of the masses was uneven but with a similar average value in all cases, approximately 51.01 ± 15.48 HU (mean ± SD) in the case series. No data were present in the literature about lesion density.

The average dimension of the lesion was 4.49 ± 2.73 cm (mean ± SD; range: 2.15–13.5 cm) for the case series and 4.70 ± 1.82 (mean ± SD; range: 2.5–8.8) for the pooled data.

All the masses had thickening of the bladder wall but, to a variable extent, 16.07 ± 10.19 (mean ± SD; range: 2–43). Similar values were observed for the depth of invasion of the organ wall, 13.14 ± 8.82 (mean ± SD; range: 5–32).

At the time of diagnosis, perivisceral extension (T3) was observed in 78.5% vs. 81.0% of cases and invasion of adjacent organs (T4) in 35.7% vs. 42.9%. Lymph node involvement was detected in 35.7% vs. 28.8% of cases and metastatic localization was found in 28.6% vs. 23.8%. The main sites of metastasis were: liver (at least six cases), bone (two cases), lung (at least one case), and brain (one case).

## 4. Discussion

Neuroendocrine carcinoma of the urinary bladder (NECB) is a rare and very aggressive tumour, which frequently manifests at an advanced stage and is identified with great difficulty, due to its infrequency. Commonly, lymph node involvement and distant metastases are already present at the time of diagnosis, which is why the average survival from the onset is rather poor [8].

This cancer is typical of Caucasian males from the 6th decade onwards, who come to observation for symptoms such as haematuria, dysuria and symptoms of the lower urinary tract (LUTS), like urinary obstruction, cystitis, pelvic pain and hydronephrosis detectable at ultrasonography of the lower abdomen [3]. These symptoms are very similar to the general onset of common urinary tract cancers [6]. Due to the aggressiveness of the disease, it is particularly important, in the presence of such symptoms, to early consider NECB in the differential diagnosis.

In the differentiation from classic urothelial cancer, the execution of a CT with scans acquired before and after intravenous administration of contrast may be relevant [26]; as a matter of fact, from the data observed in our study and from those already present in the literature, emerge some typical radiological characteristics of NECB, which are fundamental to stages of cancer and can be useful in setting up surgical treatment and therapy.

In most cases, NECB appears at CECT as a single bulky mass, protruding into the bladder lumen and with irregular margins, with a large implant base and infiltrating the organ wall. In rare cases, calcifications or necrotic areas can be observed within the mass. After intravenous administration of contrast, all lesions show enhancement, which in many cases is concentrated in the area of the wall.

As also suggested by Boyer et al. [13] and Kim et al. [12], a very frequent feature in examined cases is the presence of lymph node involvement and metastases at the onset.

Compared to data emerging from the review of literature, in our study, additional characteristics were analysed: density of the mass, morphological and contrast impregnation characteristics of the margins, thickening of the wall and depth of invasion of the bladder.

In every case covered by our study, masses appear at the onset with inhomogeneous density; margins frequently appear sharp and with a contrast agent distribution typically in the edge; furthermore, all the masses show thickening of the wall and deep invasion of the bladder structure, although with rather variable values.

These characteristics may be particularly relevant and therefore helpful in the differentiation with classic urothelial tumours, which often present with multiple intravesical localizations [27] and frequently at the onset extended to the upper urinary tract and kidneys, with resulting obstruction and hydronephrosis [28]; this relief does not occur in any of the cases investigated in our study, nor in those present in the literature.

Furthermore, classic urothelial tumour frequently has a wide diffusion of the contrast medium, due to the important neoangiogenesis, and often calcifications can be found inside the mass [29]; from our observations, these findings are much rarer in the NECB.

Therefore, during the observation of a CT scan of a patient who exhibits typical symptoms for urinary tract tumours, which are very nonspecific, it may be particularly important to focus on these differences in order to hypothesize the possible presence of an NECB, especially considering the poor survival of the patients and the need of early intervention [30].

Anyhow, radiological data must necessarily be analysed with the histological and cytological characteristics of the carcinoma [4]. Integration of all data provides the set of information necessary for setting up treatment, which in most cases consists of radical cystectomy followed by chemo and radiotherapy [3].

In our study, 130 radiomic features were analysed for the quantification of tumour phenotypic differences based on tomodensitometry (using first-order statistics), shape and texture of the lesion. The examined features presented a potential in the differentiation of NECB compared to other aggressive histological neoplasms of the bladder. Specifically, significant differences were found for the following radiomic features: dependence non-uniformity (measures the similarity of dependencies in the image: a lower value indicates more homogeneity between the dependencies in the image); Joint Average (measures the average grey level intensity of the distribution); Maximum 2D Diameter Slice (the greatest Euclidean distance on the line plane-column, usually axial, between tumour extremes); Maximum 2D Diameter Column (defined as the largest Euclidean distance in pairs between the vertices of the tumour surface mesh in the plane of the row-slice, usually coronal); Large Area Low Gray Level Emphasis (quantifies the distribution of small ones conjoined areas of low grey values within the image); Mean Absolute Deviation (the average of the distance of the density values with respect to their average value within the matrix); Bounding Box A: (the smallest possible axial dimension within which all the points of the voxel are contained); Center Of Mass B (anteroposterior distance from the center of mass of the lesion).

Although in recent years radiomic techniques have shifted attention, only one study in the literature has evaluated the radiomic characteristics of NECB, with the most significant differences found in the features “wavelet-LLH_glcm_MCC”, “wavelet-HHH_glcm_MCC”, and “wavelet-HHH_glszm_ZoneEntropy” [24]. 

In a study by Canellas et al. [31] for the prediction of the grading of pancreatic neuroendocrine neoplasms, the entropy value on CT images was predictive for the more aggressive lesions, compared to the less aggressive ones. Guo et al. [32] demonstrated that pancreatic neuroendocrine neoplasms, compared to pancreatic ductal adenocarcinomas, present a statistically greater uniformity, but a lower entropy value in the portal-venous phase with contrast medium. Conversely, no significant values for the entropy parameters were observed in our study, even though differences had been found in features associated with entropy such as dependence non-uniformity and joint average.

Similarly to our observation, in the study by Li and Colleagues [33], there were no discriminant results in kurtosis or entropy values in differentiating between pancreatic neuroendocrine tumours and pancreatic ductal adenocarcinomas. In the same study, the values concerning the first-order features (mean, median, 5th, 10th, 25th percentile) were significantly higher in neuroendocrine pancreatic tumours. However, in our study, the same parameters were not statistically significant.

Overall, the cited studies had differences from the present study, such as different imaging stages investigated (parenchymal, arterial, late), use of different software used for segmentation and feature extraction (programs developed internally, freely available open source and commercial software), and finally, several tumour sections were considered for the extraction of different features (one, three, five or all slices per lesion).

The limiting factors of our study are the intrinsic ones of the retrospective single-centre study and the small number of cases analysed; although, it must be stated that the present is one of the largest case series available in medical literature. Furthermore, the use of different types of CT scanners, which could have generated inter-scanner variability, is also limiting, just as the acquisition protocols may not be completely standardized. Lastly, the manual segmentation process is a well-known source of variability for volume-of-interest contouring. To minimize these effects, segmentations performed manually by one operator were subsequently reviewed by two other operators trained in urological imaging.

## 5. Conclusions

In conclusion, despite the scarcity of examined cases, our study shows that the suspicion of NECB must emerge at contrast-enhanced CT evaluation, especially if there is one large mass, with irregular morphology and with significant contrast enhancement, which infiltrates the bladder wall in the trigonal region and is associated with lymph node and metastatic locations in typical organs, such as bone, liver and pelvic organs. Moreover, this study presents interesting preliminary data for the radiomic characterization of bladder neuroendocrine lesions, suggesting that a radiomic signature can be identified also for NECB with non-invasive and low-cost methods, and is therefore able to differentiate the various tumour histotypes.

It would be desirable to integrate the study with prospective data, a larger cohort of patients and a longer follow-up; unfortunately, such data will be hardly obtained due to the rarity of the disease.

## Figures and Tables

**Table 1:** Demographic, clinical and imaging findings.

Author	No.	Sex	Age	Haematuria	LUTS	Hydronephrosis	RE	Location	Dimensions (cm)	Density (HU)	Margins	Necrosis	Calcifications	Wall Thickness (mm)	DOI (mm)	T3	T4	Lymphnodes	Metastasis
**Present** **study**	# 1	M	79	yes	no	yes	yes	PT	3.1 × 2.3	67	ill	no	no	11	6,5	yes	yes	no	Liver
	# 2	M	83	yes	no	yes	yes	ALT	9.5 × 4.5	30	sharp	no	no	17	32	yes	no	no	no
	# 3	M	76	yes	yes	yes	yes	ART	6.0 × 3.2	51	sharp	no	no	6	25	yes	no	no	Liver
	# 4	M	56	yes	yes	yes	yes	PRT	8.4 × 7.8	38	ill	no	no	19	21	yes	yes	yes	Bone
	# 5	M	62	yes	no	yes	no	ALRT	15.1 × 12.6	55	ill	no	yes	43	17	yes	yes	yes	no
	# 6	M	75	no	yes	yes	no	T	3.3 × 1.6	44	ill	no	no	14	5	no	no	no	no
	# 7	M	84	yes	no	yes	no	PLART	12.4 × 12.2	31	sharp	no	no	30	9,5	yes	yes	yes	no
	# 8	M	70	yes	yes	yes	no	PT	3.6 × 1.7	34	sharp	no	no	9	10	yes	yes	no	no
	# 9	M	85	yes	no	no	yes	A	4.5 × 2.0	84	ill	no	no	17	5	yes	no	no	no
	# 10	M	76	yes	no	yes	yes	PRT	3.0 × 3.3	59	ill	yes	no	15	5	no	no	yes	no
	# 11	M	83	yes	no	no	yes	PT	5.0 × 2.5	57	ill	no	no	17	13	yes	no	no	no
	# 12	M	76	yes	no	yes	no	T	2.5 × 1.9	49	ill	no	no	10	8	no	no	no	no
	# 13	M	83	yes	yes	no	yes	PR	3.0 × 1.3	68	ill	no	no	15	5	yes	no	no	no
	# 14	M	83	yes	no	no	yes	PL	2.5 × 2.5	48	ill	yes	no	2	22	yes	no	yes	Liver
**Kim** [12]	# 1	M	44					AT	3.8			yes	no			yes	yes	yes	Brain
	# 2	M	56					PLT	5.5			yes	no			yes	yes	yes	no
	# 3	M	57					AL	3			yes	no			yes	yes	yes	Liver
	# 4	M	59					AR	4.1			no	no			yes	yes	no	no
	# 5	M	66					APT	5.2			no	no			yes	yes	yes	Liver
	# 6	F	59					PT	8.2			no	yes			yes	yes	yes	no
**Author**	**No./Sex**		**Age**	**Haematuria**	**LUTS**	**Hydronephrosis**	**RE**	**Location**	**Dimensions** **(cm)**	**Density** **(HU)**	**Margins**	**Necrosis**	**Calcifications**	**Wall** **Thickness** **(mm)**	**DOI** **(mm)**	**T3**	**T4**	**Lymphnodes**	**Metastasis**
**Boyer** [13]	13 M/3 F		75.5	11/16		7/16			4.9			3/16	1/16	9 (mean)		9/16	6/16	5/16	8/16 (Liver, bone, lung)
**Xia** [14]	31 M/8 F		61.5	20/39					4.1 × 1.8				1/39			38/39		7/39	6/39
**Bote** [15]	4 M/1 F		63	5/5	2/5			T in 4/5								3/5			2/5
**Colarossi** [16]	1/F		53					P	4		sharp					yes	yes	yes	
**Prelaj** [17]	1/M		71	yes	yes		yes	A	3.4 × 2.4		ill	yes	no			no	no	no	no
**Chong** [18]	1/M		72		yes			T								yes	yes	yes	no
**Bertaccini** [19]	1/M		37	yes				P	2.5 × 2		ill	yes	no			yes	no	yes	no
**Olivieri** [20]	1/M		78	yes	yes	no	no	L	3		ill	no	no			no	no	no	no
**Praveen** [21]	1/M		50	yes	yes	yes	yes	L	3 × 3			no				yes	no	no	no
**Chekrine** [22]	1/M		84	yes		yes		PR	2.6 × 5.7		sharp	yes	no			yes	yes	yes	no
**Cerulli** [23]	1/M		60	yes	yes			T	6							yes	yes	yes	yes
**Masood** [24]	1/M		60	yes	yes			L	3 × 4								no	no	no
**He** [25]	10/M		64.9					T in 7/10	5.2			0/10				6/10	2/10	1/10	0/10

M: male; F: female; LUTS: lower urinary tract symptoms; RE: Ring enhancement; P: posterior wall; T: trigonal region; A anterior wall; L: left lateral wall; R: right lateral wall; HU: Hounsfield unit; DOI: depth of invasion; T3: Invasion of surrounding fat tissue; T4: Invasion of adjacent organs.

**Table 2:** Comparison between descriptive statistics of the case series and of the control group.

	NECB	Control	*p*-Value
**Age (years)**	76.5 ± 8.7	78.66 ± 6.60	
**Male Sex**	14/14—100.0%	29/42—69.05%	0.025
**Hydronephrosis**	10/14—71.43%	14/42—33.33%	0.027
**Haematuria**	13/14—92.86%	7/42—16.67%	
**LUTS**	5/14—35.71%	19/42—45.24%	
**density (HU)**	51.01 ± 15.48	76.27 ± 22.26	<0.001
**Product of dimensions (cm^2^)**	38.1 ± 59.3	14.44 ± 12.98	0.033
**Trigonal region involvement**	11/14—78.57%	8/42—19.05%	<0.001
**Ring enhancement**	9/14—64.29%	28/42—66.67%	
**Ill-defined margins**	10/14—71.43%	29/42—69.05%	
**Intra-mass necrosis**	2/14—14.29%	11/42—26.19%	
**Calcifications**	1/14—7.14%	2/42—4.76%	
**cT3**	3/14—21.43%	33/42—78.57%	
**cT4**	5/14—35.71%	22/42—52.38%	
**Lymph-node involvement**	5/14—35.71%	23/42—54.76%	
**Distant metastasis**	4/14—28.57%	10/42—23.81%	

## Data Availability

Data is contained within the article or supplementary material.

## Supplementary Materials

The following supporting information can be downloaded at: https://www.mdpi.com/article/10.3390/jcm12206510/s1, File S1: List of shape-based features and first-order features; File S2: List of texture features.
