# Supplementary material for: Neuroendocrine Carcinoma of the Urinary Bladder: CT Findings and Radiomics Signature
Source: J Clin Med. 2023 Oct 13;12(20):6510. doi: 10.3390/jcm12206510 (PMC10607129; doi:10.3390/jcm12206510)
Supplement: Supplementary file 1 [file jcm-12-06510-s001.zip › jcm-2650762-supplementary.pdf]

Journal of
Clinical Medicine

MDPI

Supplementary Materials:

SHAPED-BASED FEATURES	FIRST-ORDER FEATURES
Flatness	10Percentile
Leastaxislength	90Percentile
Majoraxislength	Energy
Maximum2Ddiametercolumn	Entropy
Maximum2Ddiameterrow	InterquartileRange
Maximum2Ddiameterslice	Kurtosis
Maximum3Ddiameter	Maximum
Meshvolume	MeanAbsoluteDeviation
Minoraxislength	Mean
Sphericity	Median
Surfacearea	Minimum
Surfacevolumeratio	Range
Voxelvolume	RobustMeanAbsoluteDeviation
	RootMeanSquared
	Skewness
	TotalEnergy
	Uniformity
	Variance

Supplementary Material File S1. List of shape-based features and first-order features.

SECOND-ORDER FEATURES	
Autocorrelation	GrayLevelNonUniformity
ClusterProminence	GrayLevelNonUniformityNormalized
ClusterShade	GrayLevelVariance
ClusterTendency	HighGrayLevelRunEmphasis
Contrast	LongRunEmphasis
Correlation	LongRunHighGrayLevelEmphasis
DifferenceAverage	LongRunLowGrayLevelEmphasis
DifferenceEntropy	LowGrayLevelRunEmphasis
DifferenceVariance	RunEntropy
Id	RunLengthNonUniformity
Idm	RunLengthNonUniformityNormalized
Idmn	RunPercentage RunVariance
Idn	ShortRunEmphasis
Imc1	ShortRunHighGrayLevelEmphasis
Imc2	ShortRunLowGrayLevelEmphasis
InverseVariance	GrayLevelNonUniformity
JointAverage	GrayLevelNonUniformityNormalized
JointEnergy	GrayLevelVariance
JointEntropy	HighGrayLevelZoneEmphasis
MCC	LargeAreaEmphasis
MaximumProbability	LargeAreaHighGrayLevelEmphasis
SumAverage	LargeAreaLowGrayLevelEmphasis
SumEntropy	LowGrayLevelZoneEmphasis
SumSquares	SizeZoneNonUniformity
DependenceEntropy	SizeZoneNonUniformityNormalized
DependenceNonUniformity	SmallAreaEmphasis
DependenceNonUniformityNormalized	SmallAreaHighGrayLevelEmphasis
DependenceVariance	SmallAreaLowGrayLevelEmphasis
GrayLevelNonUniformity	ZoneEntropy
GrayLevelVariance	ZonePercentage
HighGrayLevelEmphasis	ZoneVariance
LargeDependenceEmphasis	Busyness
LargeDependenceHighGrayLevelEmphasis	Coarseness
LargeDependenceLowGrayLevelEmphasis	Complexity
LowGrayLevelEmphasis	Contrast
SmallDependenceEmphasis	Strength
SmallDependenceHighGrayLevelEmphasis	

Supplementary Material File S2. List of texture features.
